# Supplementary material for: Production and Characterization of Cellulose Nanocrystals from Eucalyptus Dissolving Pulp Using Endoglucanases from Myceliophthora thermophila
Source: Int J Mol Sci. 2023 Jun 26;24(13):10676. doi: 10.3390/ijms241310676 (PMC10341860; doi:10.3390/ijms241310676)
Supplement: Supplementary file 1 [file ijms-24-10676-s001.zip › ijms-2452907-supplementary.pdf]

Production and characterization of Cellulose nanocrystals from Eucalyptus dissolving pulp using endoglucanases from *Myceliophthora thermophila*

Pratima Waghmare, Nuo Xu, Pankajkumar Waghmare, Goudong Liu, Yinbo Qu, Xuezhi Li, Jian Zhao*

State Key Laboratory of Microbial Technology, Shandong University, Qingdao 266237, China

* Corresponding author, Email: zhaojian@sdu.edu.cn (J. Zhao)

Table S1. Primers (gene-specific) for cassette construction. The His6-tag encoding sequences were underlined

PubiD-F	CTTCTCGCTACCCATCTCTTTC
PubiD-R	CAACTCCGGAGCTGACATCG
PubiD-MtEG5A-F	CTTACGACTGCCGTCACCATGAAGTCCTCCATCCTCG
PubiD-MtEG5A-R	GTAACGTTAAGTGGATCTTAGTGGTGGTGGTGGTGGTGCGGCAAGTACTTCTTCAAG
PubiD-MtEG7A-F	CTTACGACTGCCGTCACCATGGGGCAGAAGACTCTCC
PubiD-MtEG7A-R	GTAACGTTAAGTGGATCCTAGTGGTGGTGGTGGTGGTG CAGGCACTGCGAGTAC
PubiD-MtEG12A-F	CTTACGACTGCCGTCACC ATGCAGCCGTTTCTGCTCTT
PubiD-MtEG12A-R	GTAACGTTAAGTGGATCTTAGTGGTGGTGGTGGTGGTG AGCAACCCTTGCAGAGTA
PubiD-MtEG45A-F	CTTACGACTGCCGTCACC ATGCATCTCTCCGCCACC
PubiD-MtEG45A-R	GTAACGTTAAGTGGATCTTAGTGGTGGTGGTGGTGGTG ACGGGAGCAGCCGCT

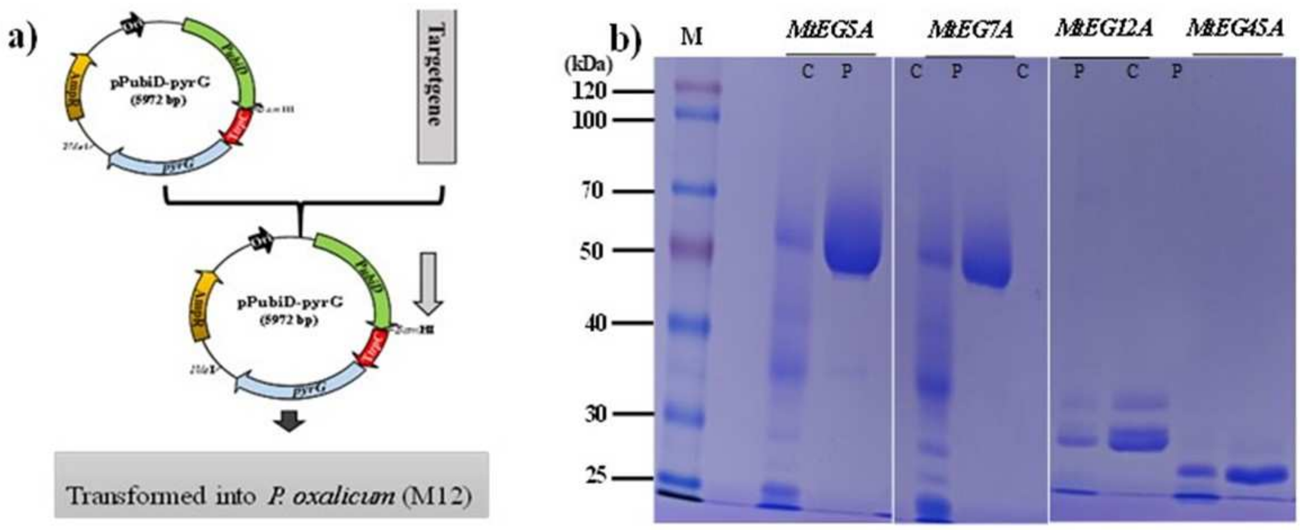

Figure S1. Schematic diagram of strain construction (a) and SDS PAGE analysis of crude and purified recombinant endoglucanases (b). In the SDS-PAGE, C and P refer to crude enzyme and purified endoglucanases, respectively.

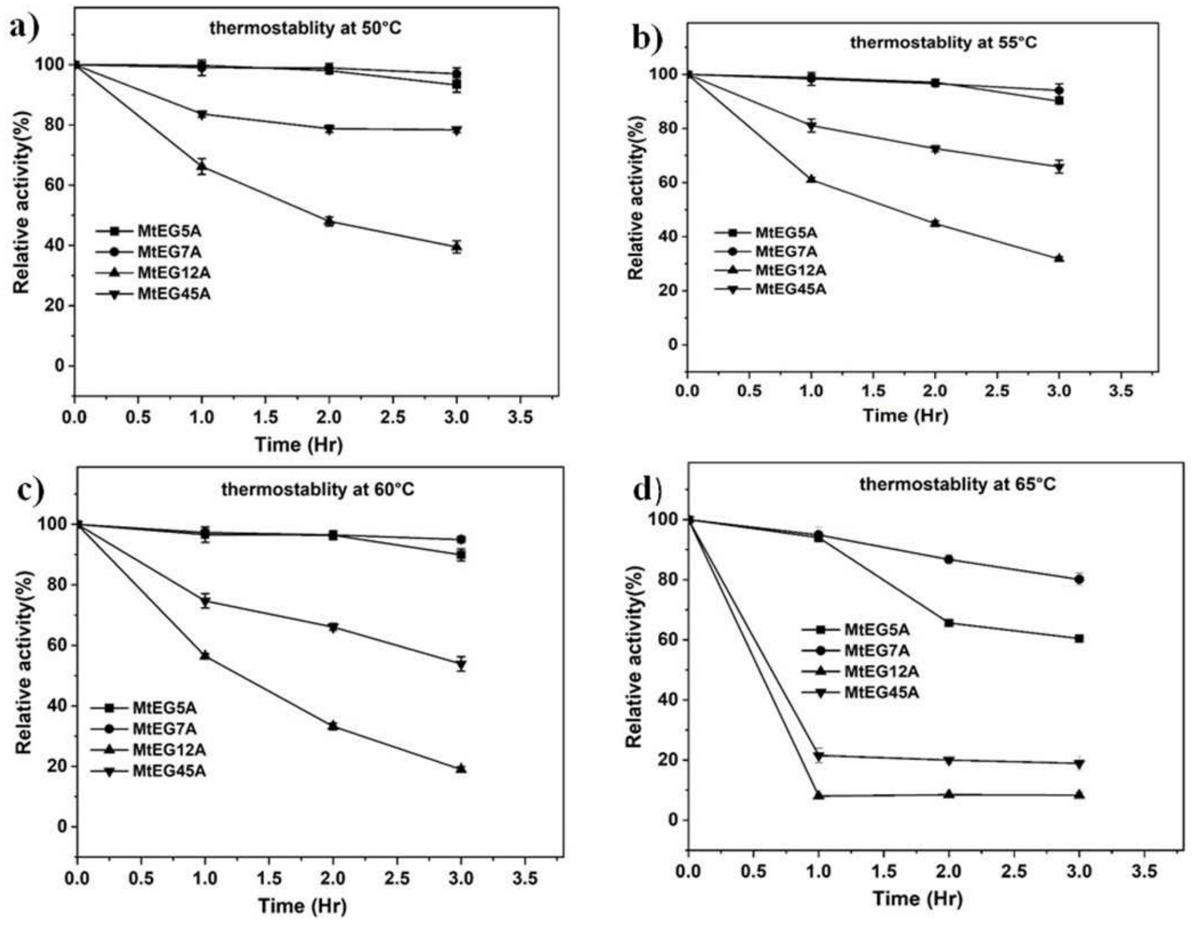

Figure S2. Thermostability of purified recombinant endoglucanases at 50°C(a), 55°C (b), 60 °C (c), and 65°C (d).

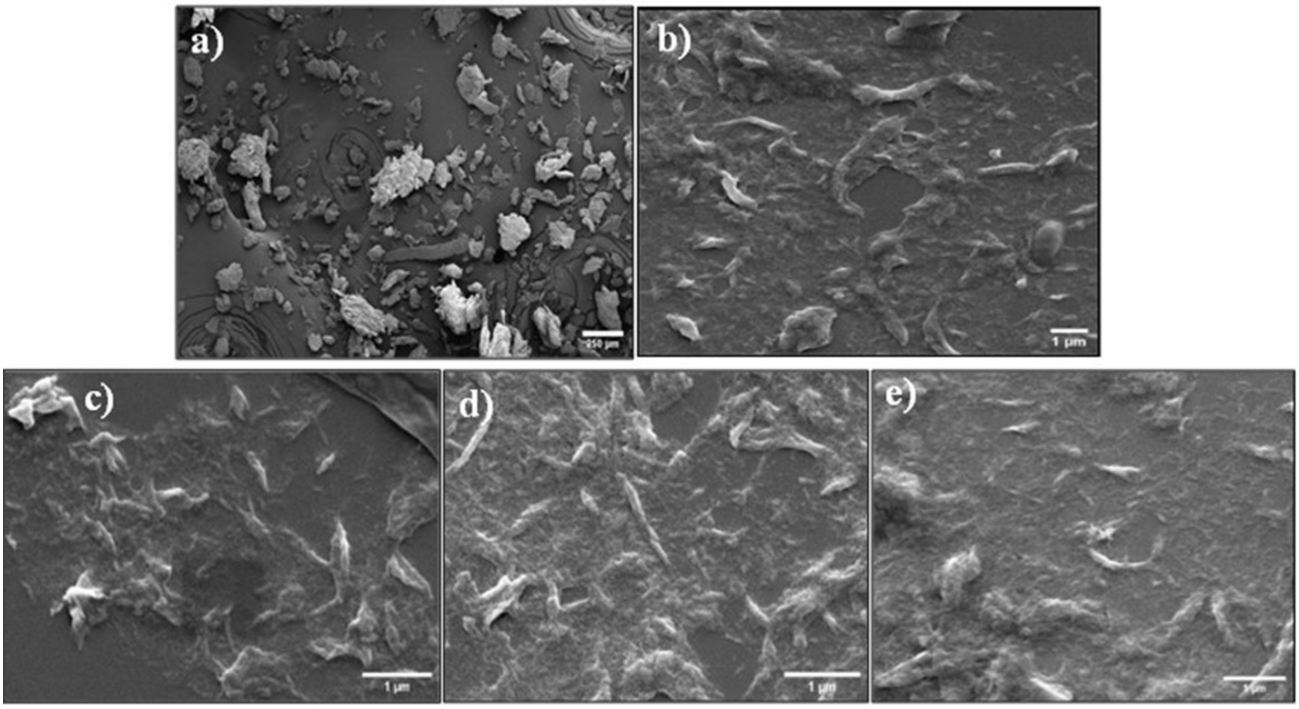

Figure S3. SEM images of EDP (a) and CNCs prepared by enzymatic hydrolysis of EDP using MtEG5A (b), (5+7) (c), (5+12) (d), and (5+7+45) (e), respectively.

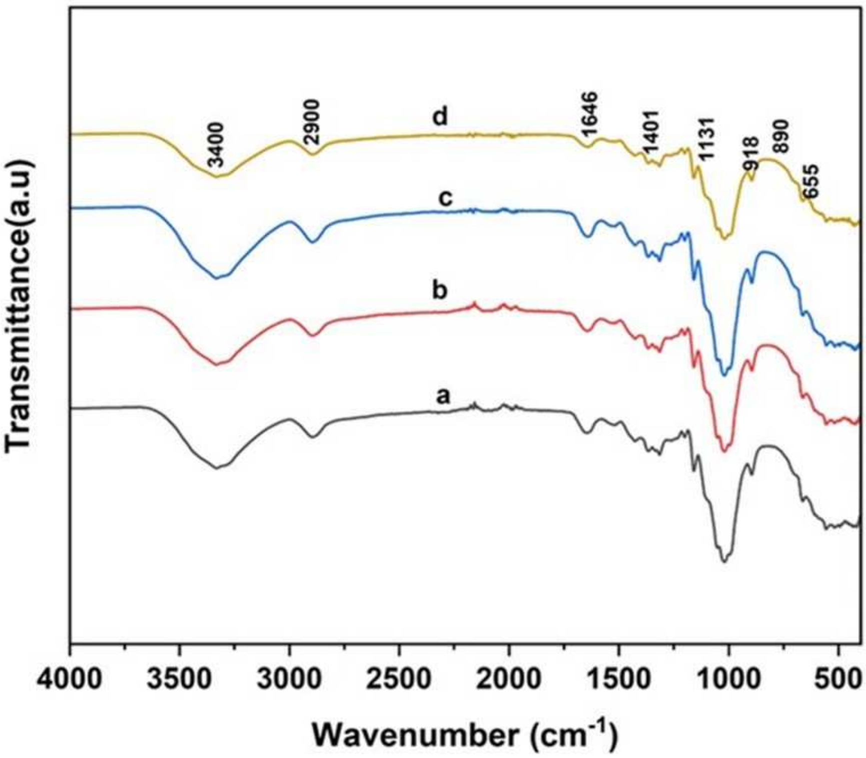

Figure S4. FTIR of CNCs prepared by enzymatic hydrolysis of EDP using MtEG5A (a); (5+7) (b); (5+12) (c); and (5+7+45) (d), respectively.
